# Patient pathways, presentation time, time to diagnosis, and early survival outcomes in pediatric acute lymphoblastic leukemia at Muhimbili National Hospital, Dar es Salaam, Tanzania

**DOI:** 10.3389/fped.2026.1777603

**Published:** 2026-04-01

**Authors:** Ruchius Philbert, Hadija Mwamtemi, Tom Okade, Huzaifah Mutyaba, Lilian Ndyetabula, Esther Shimba, Nana Jacqueline Nakiddu, Emmanuel Elisha, Wende Clarence Safari, Faraja Chiwanga, Lulu Chirande

**Affiliations:** 1 Department of Paediatrics and Child Health, Muhimbili National Hospital, Dar-es-salaam, Tanzania; 2 Department of Paediatrics and Child Health, Muhimbili National Hospital, Dar-es-salaam, Tanzania; 3 Makerere University School of Public Health, Kampala, Uganda; 4 Tumaini La Maisha Organization, Dar-es-salaam, Tanzania; 5 Clinical Research, Training and Consultancy Unit, Muhimbili National Hospital, Dar-es-salaam, Tanzania; 6 Muhimbili University of Health and Allied Sciences, Dar -es-salaam, Tanzania; 7 Department of Paediatrics and Child Health, Muhimbili University of Health and Allied Sciences, Dar-es-salaam, Tanzania; 8 Department of Health Services Research and Policy, London School of Hygiene and Tropical Medicine (LSHTM), London, United Kingdom; 9 Directorate of Medical Services, Muhimbili National Hospital, Dar es Salaam, Tanzania

**Keywords:** acute lymphoblastic leukemia, patient pathways, presentation time, survival, time to diagnosis

## Abstract

**Background:**

The Tanzanian healthcare system is complex, and weaknesses in referral pathways for time-sensitive conditions such as cancer contribute to poor outcomes among pediatric oncology patients. Early presentation and timely diagnosis are essential for improving survival in pediatric leukemia; however, evidence describing delays within Tanzania's referral system and their effects on early survival in children with acute lymphoblastic leukemia (ALL) remains limited. This study addresses this gap by characterizing patient pathways, estimating time to presentation and diagnosis, and assessing early survival among children with ALL treated at Muhimbili National Hospital (MNH).

**Methods:**

We conducted a hospital-based retrospective observational cohort study among 64 pediatric patients with a confirmed diagnosis of ALL admitted to the Pediatric Oncology Unit of Muhimbili National Hospital between January and December 2024. Descriptive statistics were used to summarize patient pathways, presentation time, and diagnosis. Six- and twelve-month survival probability was estimated using Kaplan–Meier methods, and survival outcome differences were assessed using the log-rank test.

**Results:**

Sixty-four children with ALL were included; more than half (67.2%) were boys, and the median age at diagnosis was 6 years. Nearly half of the children (46.9%) were aged 0–5 years. Most children (51.6%) first sought care at primary-level facilities, whereas only 18.8% were referred from secondary-level facilities. The median time from symptom onset to presentation at MNH was 28.5 days, followed by a median time to diagnosis of 14 days. Six- and 12-month survival probabilities were 79% and 72%, respectively. Survival did not differ significantly by gender (log-rank *χ*^2^ = 0.24, *p* = 0.63), age group (*χ*^2^ = 2.01, *p* = 0.57), presentation time (*χ*^2^ = 0.79, *p* = 0.37), time to diagnosis [*χ*^2^(1) = 0.04, *p* = 0.84], or referral pathway length (*χ*^2^ = 0.11, *p* = 0.74).

**Conclusion:**

Overall, delays in presentation and referral remain significant barriers in the management of childhood ALL in this setting. Most mortality occurred during the early treatment period, highlighting the critical importance of strengthening supportive care, early complication management, and infection control alongside improvements in referral systems. Efforts to strengthen early detection and build capacity at lower-level health facilities may substantially improve survival among children with ALL in Tanzania and other low-resource settings.

## Introduction

Childhood cancer is one of the leading causes of mortality among children and adolescents aged 0–19 years (1). Tanzania is among the sub-Saharan African countries that have prioritized cancer care (2). Every year, an estimated 400,000 children and adolescents are newly diagnosed with childhood cancer globally, with more than 90% of cases occurring in low- and middle-income countries (LMICs) (3). The likelihood of surviving a childhood cancer diagnosis varies significantly across countries. The literature indicates that over 80% of children with cancer in high-income countries achieve successful treatment and cure, whereas in many LMICs, fewer than 30% survive (4, 5). Avoidable deaths in LMICs are largely attributable to factors such as delayed or missed diagnosis, misdiagnosis, limited access to care, treatment abandonment, therapy-related complications, and disease relapse (4, 5). Although LMICs account for a higher incidence of childhood cancer cases annually, the 5-year survival probability is substantially lower that 20% (6). The main contributors to reduced survival rates are late presentation, malnutrition, and suboptimal supportive and intensive care facilities, resulting in high treatment-related mortality (7).

The World Health Organization's Global Initiative for Childhood Cancer aims to achieve a survival rate of at least 60% for children with cancer worldwide by 2030 (8, 9). This initiative seeks to reduce global disparities in childhood cancer outcomes, recognizing that survival now exceeds 90% in many high-income countries but remains below 30% in numerous LMICs (8, 9). In Tanzania, Tumaini La Maisha (TLM), a non-governmental organization (NGO) dedicated to supporting children with cancer, estimates that approximately 4,500 children develop cancer every year (10), underscoring the significant burden of childhood cancer in Tanzania. The most common pediatric cancers include leukemias, brain tumors, lymphomas, and solid tumors such as neuroblastoma and Wilms tumor (1). Acute lymphoblastic leukemia (ALL) is the most common childhood cancer globally and in Tanzania. Although ALL predominantly affects children, it can occur in adults as well, and it accounts for 80% of all leukemia cases in children (11).

Long-term cure rates for childhood ALL now exceed 90% in high-income countries (HICS) (12). However, outcomes for children and adolescents with cancer in LMICs remain poor. Limited access to appropriate treatments and delay in diagnosis continue to challenge, contributing to the observed disparities in outcome between HICs and LMICs. The one-year overall survival rate for pediatric patients with ALL in HICs exceeds 95%, reflecting significant advances in the supportive and risk-adapted care (13). Despite the existing wider survival inequality between HICs and LMICs, Muhimbili as a tertiary-level hospital in Tanzania has adopted different multidimensional approaches to improve cancer care for children (12). However, delays in presentation to appropriate treatments sites and delays in diagnosis may occur throughout the diagnostic pathway. Early diagnosis of childhood cancer is crucial to reduce mortality (14). For example, a study conducted in Bangladesh reported a median diagnostic delay of 60 days, highlighting how prolonged delays can allow potentially treatable cancers to progress and become more difficult to manage (15).

Patient pathways refer to the sequence of healthcare facilities visited by a patient from the onset of symptoms to arrival at a specialized treatment center. Tanzania’s health system is structured into three levels: primary level (dispensaries, health centers, and district hospitals), secondary level (regional referral hospitals), and tertiary level (national hospitals and zonal referral hospitals). Weak referral pathways between primary and specialized cancer units contribute to significant delays that negatively affect treatment outcomes (16). Additional health system challenges include limited resources and capacity, misdiagnosis, delayed diagnosis, lack of childhood cancer awareness in lower-level facilities (primary level), absence of universal health insurance coverage, and poor coordination across levels of care.

Nonetheless, there is growing evidence that improvements are possible. Short referral pathways, early presentation to hospitals, and timely diagnosis could improve both survival and outcomes for patients. This study therefore assessed the guardian's pathways from the initial sign of disease, presentation time to a specialized oncological center, and time to diagnosis among the pediatric ALL patients admitted to Muhimbili National Hospital between January and December 2024. Findings from this study could inform targeted strategies to ensure easier accessibility of appropriate treatment and timely diagnosis, enabling earlier initiation of cancer treatment to achieve positive outcomes among pediatric ALL patients in Tanzania and similar resource-limited settings.

## Materials and methods

### Study design

This was a hospital-based retrospective observational cohort study conducted among pediatric patients with a confirmed diagnosis of acute lymphoblastic leukemia admitted to the Pediatric Oncology Unit of Muhimbili National Hospital between January and December 2024. Children diagnosed with ALL during this period were observed for up to 12 months to examine both 6- and 12-month survival outcomes. This study received ethical approval from the Muhimbili National Hospital Institutional Review Board (MNHIRB) at the Clinical Research/Training/Consultancy Unit (MNH/CRTCU/Perm/2025/255). The requirement for informed consent and assent was waived by MNHIRB due to the retrospective nature of the study.

### Study setting

Muhimbili National Hospital is a national tertiary referral hospital located on the eastern coast of Tanzania, serving a substantial number of patients across the country. The study was conducted at the pediatric cancer unit of MNH, which has a capacity of 78 beds and is staffed by four pediatricians, five pediatric oncology specialists, five registrars, four pharmacists, and 29 nurses (17).

### Study population

The study population included pediatric cancer patients (aged 0–19 years) with a confirmed diagnosis of ALL, who were undergoing chemotherapy treatment at the oncology unit at MNH between January and December 2024. Hospital registry data and patients’ clinical notes were reviewed to understand their history of admission (pathways), presentation time to appropriate treatment sites, and time to diagnosis for those diagnosed with ALL.

### Diagnosis and treatment

Children suspected of having leukemia underwent a clinical assessment that included history taking, physical examination, and laboratory investigations. Bone marrow aspirate or biopsy, peripheral blood smear, and cerebrospinal fluid (CSF) samples were collected and processed at the MNH laboratory. Diagnosis was confirmed by flow cytometry of peripheral blood smear or bone marrow aspirate, performed either at MNH or a nearby private laboratory. Results and risk group information were recorded in both paper-based files and electronic records. Treatment followed a modified UK ALL protocol for confirmed ALL cases. A 7-day prednisolone prephase was administered prior to initiation of multi-agent chemotherapy as part of standard induction therapy.

### Selection criteria

#### Inclusion criteria

All pediatric patients with a confirmed diagnosis of ALL admitted between January 2024 and December 2024 and receiving chemotherapy or other adjuvant cancer drugs were included. Complete medical records with documented dates of symptom onset, presentation, diagnosis, and early outcome follow-up were required.

#### Exclusion criteria

Patients diagnosed at other facilities and referred to Muhimbili National Hospital after initiation of chemotherapy, patients with incomplete records lacking key dates (symptom onset, hospital presentation, or diagnostic confirmation), and patients with relapsed ALL.

### Sample size and sampling technique

The study employed a census method, whereby all children with a confirmed diagnosis of ALL were included, as the number was manageable in terms of time and available resources.

In 2024, a total of 449 children were diagnosed with cancer at MNH, of whom 113 had acute leukemia. Among these, 64 were confirmed to have acute lymphoblastic leukemia. Leukemia was the most common childhood cancer diagnosed at MNH during the study period compared with other pediatric malignancies. All 64 children with confirmed ALL met the inclusion criteria and were therefore enrolled in the study. Children diagnosed with ALL were monitored from the date of diagnosis to assess early survival outcomes. Follow-up information was obtained from hospital registries, clinical files, and phone interviews with parents or guardians, where necessary, to ascertain survival status.

### Study procedures

With the support of four trained research assistants with health-related backgrounds, we reviewed the pediatric oncology and cancer registry databases at MNH to identify all children aged 0–14 years who were diagnosed with ALL between January and December 2024. The list obtained from the registry was cross-checked against pediatric oncology ward admission registers to ensure completeness and accuracy. Medical records and clinical files of all eligible children were then retrieved. Using a structured data abstraction tool, information was extracted on sociodemographic characteristics, clinical presentation, patient pathways, presentation time, referral history, and time to diagnostic confirmation of ALL. Dates related to symptom onset, first health facility contact, admission to MNH, and diagnostic confirmation were carefully recorded to allow computation of time intervals. In addition, laboratory and pathology records were reviewed to confirm the diagnosis of ALL based on bone marrow examination, with immunophenotyping results included where available. Clinical findings at presentation and relevant laboratory parameters were also documented. To assess early survival outcomes, children diagnosed with ALL in 2024 were monitored for up to 6 months and subsequently up to 12 months from the date of diagnosis. Survival status and dates of death, where applicable, were obtained from hospital records. For patients whose survival status could not be confirmed from hospital files alone, telephone interviews with parents or guardians were conducted using contact information available in the medical records. During these interviews, caregivers were asked to confirm the child's vital status and date of death if the child had died.

### Study variables

The dependent variable was early survival outcome among children diagnosed with ALL, assessed as survival status and time to event within the follow-up period. The independent variables included sociodemographic characteristics (age and sex), patient pathway factors (first point of healthcare contact, referral pathways, levels of health facilities visited prior to admission at Muhimbili National Hospital, and intra-hospital referral), and time-related factors (presentation time and time to diagnosis).

### Data management and analysis

Data were entered into Microsoft Excel 2016 for cleaning and exported to STATA 17.0 software for analysis. Continuous variables (age, presentation time, time to diagnosis) were summarized using means, medians, and ranges, while categorical variables (sex, referral pathways) were summarized as frequencies and percentages. Survival analysis was performed using the Kaplan–Meier method, with curves generated for the overall cohort and stratified by sex, age group, presentation time, time to diagnosis and referral pathways. Differences in survival outcomes were compared using the log-rank test. Survival time was calculated from the date of diagnosis to death or last follow-up. Statistical significance was set at *p* < 0.05.

## Results

### Sociodemographics, presentation time, time to diagnosis, and survival outcomes

A total of 64 participants aged 0–19 years met the inclusion criteria and were enrolled in the study. The majority were boys (67.2%). Almost half of the participants (46.9%) were in the 0–5 year age group, followed by the 5–10 year age group, which accounted for 31.2%. The median age of participants was 6 years, and the mean age was 6.8 ± 5.0 years. In the context of this study, presentation time referred to the length of time between the initial onset of signs and symptoms of childhood cancer and the patient's first visit to the oncology center for specialized treatment. It has been demonstrated that presentation time plays a crucial and decisive role in cancer treatment outcomes. This study found that the median presentation time was 28 days, with an average of 40.7 days.

Time to diagnosis was defined as the duration from the patient's arrival at the oncology center to the confirmation of a cancer diagnosis. The diagnosis of ALL typically begins with a medical history, physical examination, complete blood count, and peripheral blood smears, with bone marrow examination providing definitive confirmation. In this study, the median time to diagnosis was 14 days, with an average of 28.5 days. Survival outcome was defined as the clinical status of pediatric ALL patients admitted between January and December 2024. At follow-up, 68.8% of the patients were alive, while 31.2% had died (Table 1).

**Table 1:** Demographics, presentation time, time to diagnosis, and outcomes (*N* = 64).

Category	Subcategory	Frequency (*n* = 64)	Percentage (%)
Age group (years)	0–5	30	46.9
	>5–10	20	31.2
	>10–15	8	12.5
	>15–20	6	9.4
Age summary (years)	Mean	6.8	
	Median	6.0	
	Standard deviation	5.0	
	Minimum	0.0	
	Maximum	20.0	
Gender	Male	43	67.2
	Female	21	32.8
Presentation time (days)	Mean	40.7	
	Median	28.5	
	Standard deviation	37.3	
	Minimum	2.0	
	Maximum	141.0	
Time to diagnosis (days)	Mean	16.0	
	Median	14.0	
	Standard deviation	8.4	
	Minimum	7.0	
	Maximum	31.0	
Survival outcome	Alive	44	68.8
	Died	20	31.2

### Patient pathways

In this study, pathways referred to the levels of healthcare facilities visited by patients before reaching the specialized oncology unit for cancer treatment at MNH. The findings show that the majority of patients who attended secondary-level facilities (regional hospitals) were referred directly to the MNH Oncology Unit, accounting for 18.8%. This was followed by 17.2% of patients who passed through primary-level facilities (dispensaries), then secondary-level facilities, before arriving at the MNH Oncology Unit.

In addition, caregivers were asked to identify each health facility visited, including the number of days spent at each level before reaching the specialized oncology unit at MNH. Facility types were categorized based on guidelines of the Tanzanian Ministry of Health, Community Development, Gender, Elderly and Children. The majority of participants reported that their first point of contact was at a primary-level facility, where most delays are hypothesized to occur. These delays are believed to contribute to disparities in survival outcomes between developed and developing countries. Almost half of the participants (51.6%) first attended primary-level facilities.

This study also identified delays within the national hospital, with 31.3% of participants initially admitted to non-oncology units at Muhimbili National Hospital (MNH) before being transferred to the paediatric oncology unit, reflecting intra-hospital delays. In addition, 29.9% of patients were first managed at zonal hospitals prior to referral to the paediatric oncology unit at MNH. These zonal hospitals included Bugando Medical Centre, Benjamin Mkapa Hospital, Mbeya Zonal Hospital, and Kilimanjaro Christian Medical Centre (KCMC) (Table 2).

**Table 2:** Referral pathways to the MNH-Oncology Unit prior to admission (*N* = 64).

Referral pathway	Percentage (%)
Home → Secondary level facility → MNH-oncology unit	18.8
Home → Primary level facility → Secondary level facility → MNH-oncology unit	17.2
Home → Primary level facility → Secondary level facility → MNH_non-oncology unit → MNH-oncology unit	15.6
Home→Secondary level facility → Zonal referral hospital → MNH-oncology unit	12.5
Home → Secondary level facility → MNH_non-oncology unit → MNH-oncology unit	12.5
Home → Primary level facility → Secondary level facility → Zonal referral hospital → MNH-oncology unit	4.7
Home → Primary level facility → Zonal referral hospital → MNH-oncology unit	3.1
Home → Primary level facility → Secondary level facility → MNH-oncology unit	3.1
Home → Primary level facility → MNH-oncology unit	3.1
Home → Secondary level facility → Zonal referral hospital → MNH_non-oncology unit → MNH-oncology unit	1.6
Home → Primary level facility → Zonal referral hospital → MNH-oncology unit	1.6
Home → Primary level facility → Secondary level facility → Zonal referral hospital → MNH-oncology unit	1.6
Home → Zonal referral hospital → MNH-oncology unit	1.6
Home → Zonal referral hospital → MNH_non-oncology unit → MNH-oncology unit	1.6
Home → Primary level facility → Zonal referral hospital → MNH_non-oncology unit → MNH-oncology unit	1.6

### Survival probability of pediatric patients

**Figure 1:**
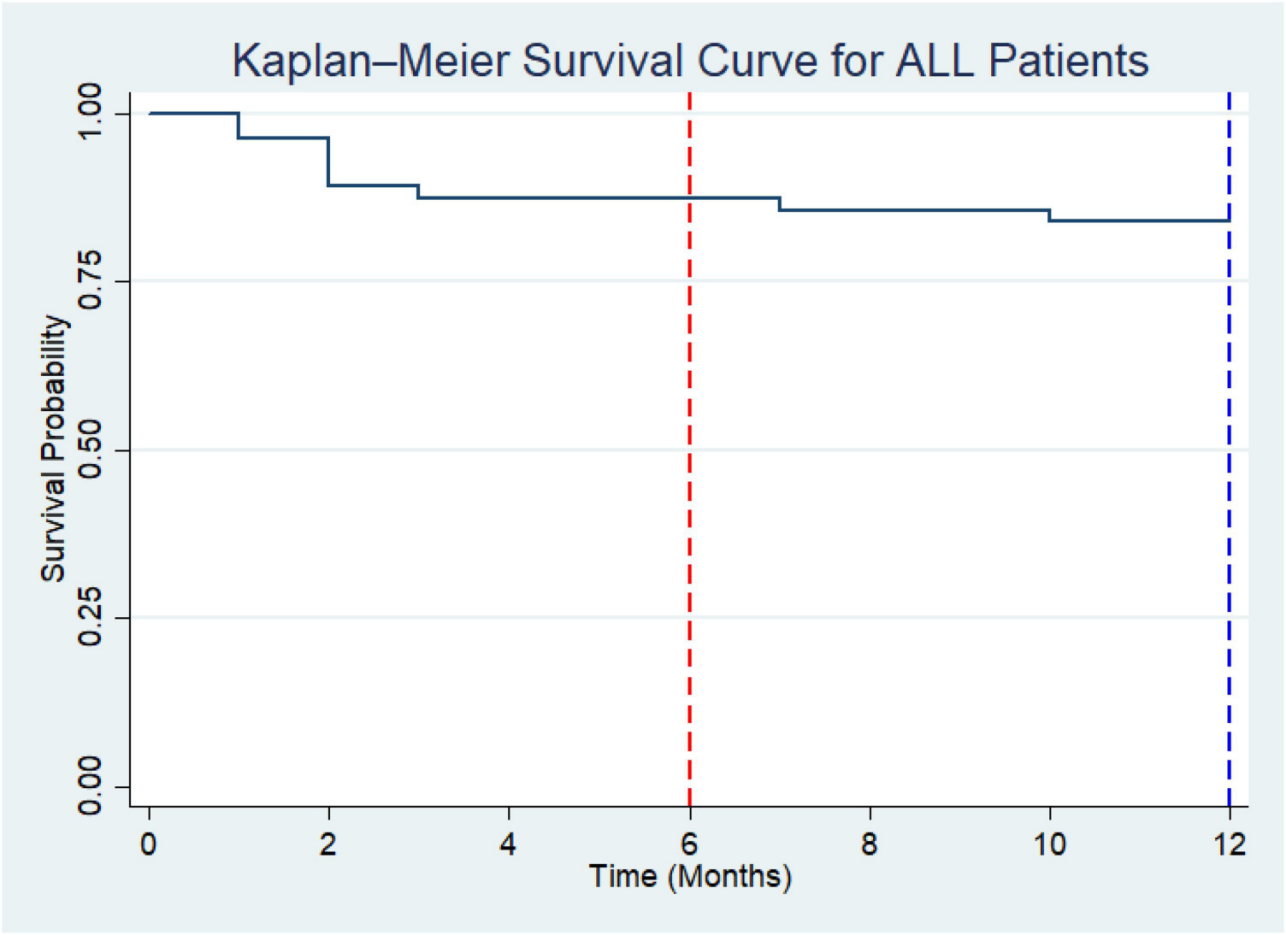
Kaplan–Meier survival curve for ALL patients (overall) (*N* = 44).

The overall Kaplan–Meier survival analysis showed that the median survival time was not reached during the follow-up period, indicating that more than half (68.8%) of patients remained alive by the end of the observation period. The survival probability at 6 months was 79% (95% CI: 63%–88%), and at 12 months, it was 72% (95% CI: 55%–84%), demonstrating a gradual decline in survival over time (Figure 1).

### Survival analysis by gender

**Figure 2:**
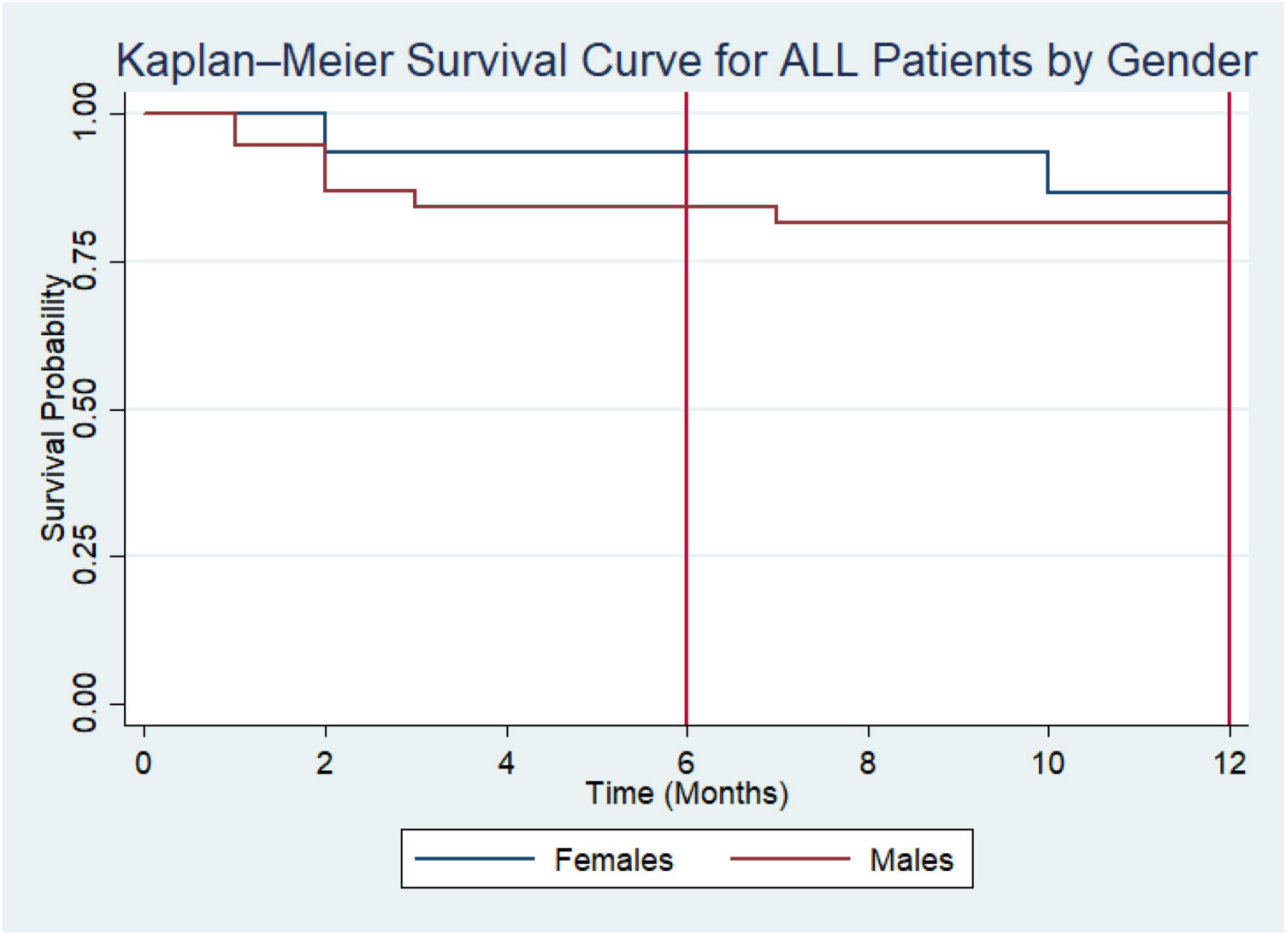
Kaplan–Meier survival curves for ALL patients stratified by gender.

Kaplan–Meier survival estimates stratified by gender revealed that the median survival time was not reached for either male or female patients during the follow-up period, indicating that more than half (50%) of patients in both groups remained alive at the end of the observation period. At 6 months, the survival probability was 84.2% (95% CI: 68.2%–92.6%) for boys and 93.3% (95% CI: 61.3%–99.0%) for girls. At 12 months, survival probability declined slightly to 81.6% (95% CI: 65.2%–90.8%) in boys and 86.7% (95% CI: 56.4%–96.5%) in girls. Although girls demonstrated higher survival probabilities at both 6 and 12 months, the overlapping confidence intervals indicated that this difference was not statistically significant (log-rank *χ*^2^ = 0.03, *p* = 0.63) (Figure 2).

### Survival analysis by age group

**Figure 3:**
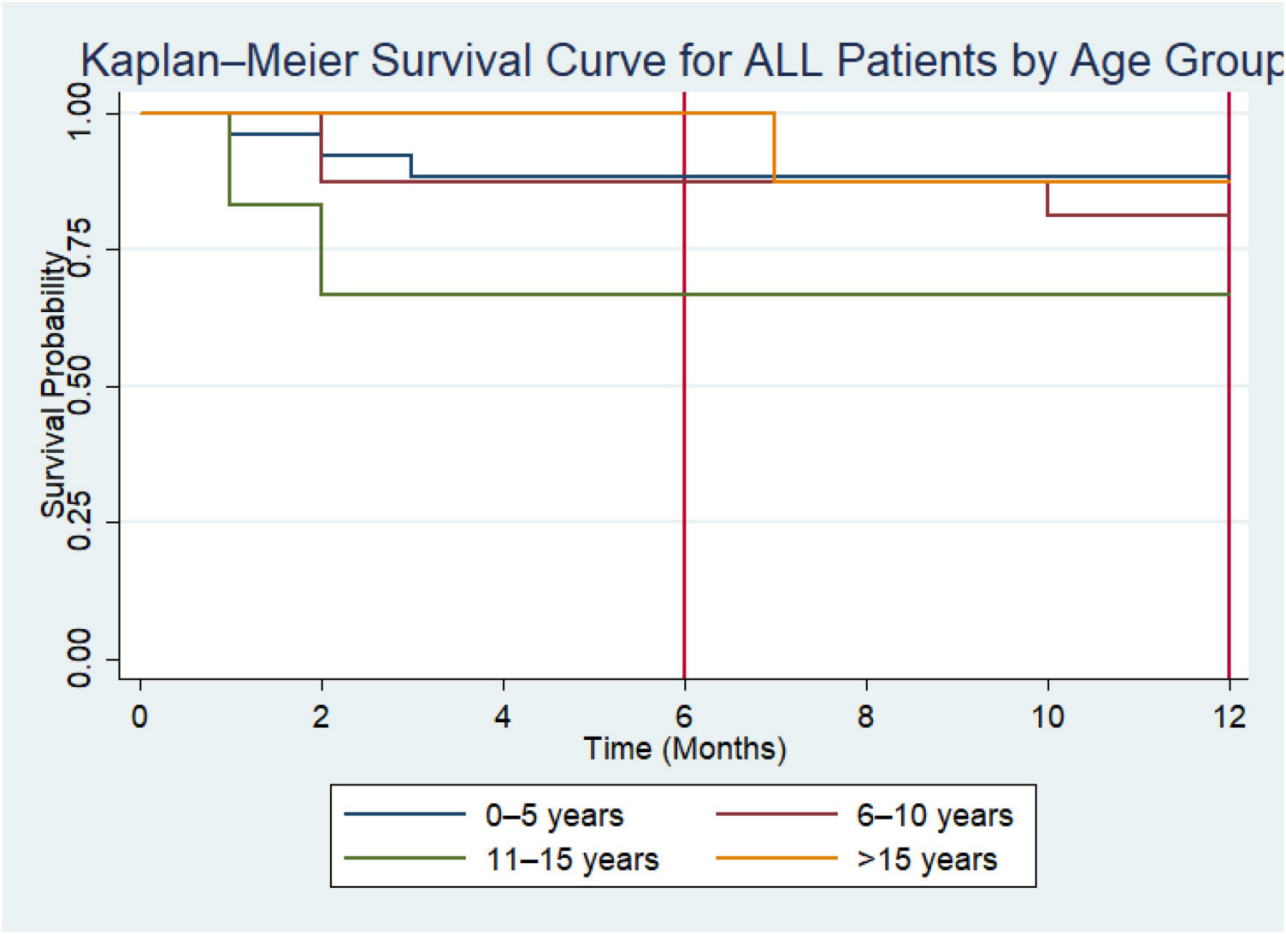
Kaplan–Meier survival curves for ALL patients stratified by age group.

Kaplan–Meier survival curves stratified by age group demonstrated that survival probability remained above 50% throughout the 12-month follow-up period in all categories; therefore, the median survival time was not reached for any age group. At 6 months, survival probability was 88.5% (95% CI: 68.4%–96.1%) among children aged 0–5 years and 87.5% (95% CI: 58.6%–96.7%) among those aged 6–10 years. Survival was comparatively lower among children aged 11–15 years at 66.7% (95% CI: 19.5%–90.4%). No deaths were observed in the >15-year group before 6 months. At 12 months, survival probability remained 88.5% (95% CI: 68.4%–96.1%) in the 0–5-year group and declined slightly to 81.3% (95% CI: 52.5%–93.5%) in the 6–10-year group. Survival in the 11–15-year group remained 66.7% (95% CI: 19.5%–90.4%), while children aged >15 years had a survival probability of 80.0% (95% CI: 20.4%–96.9%). Although children aged 11–15 years appeared to have comparatively lower survival probabilities, the confidence intervals overlapped substantially across groups. The log-rank test showed no statistically significant difference in survival distributions by age category [*χ*^2^(3) = 2.01, *p* = 0.57] (Figure 3).

### Survival analysis by presentation time

Kaplan–Meier survival estimates stratified by presentation time demonstrated that survival probability remained above 50% throughout the 12-month follow-up period in both groups; therefore, the median survival time was not reached for either group. At 6 months, the survival probability was 82.4% (95% CI: 54.7%–93.9%) among children who presented within 28 days of symptom onset, compared with 88.9% (95% CI: 73.1%–95.7%) among those who presented after 28 days. At 12 months, survival probability declined to 76.5% (95% CI: 48.8%–90.5%) in the ≤28-day group and to 86.1% (95% CI: 69.8%–93.9%) in the >28-day group.

**Figure 4:**
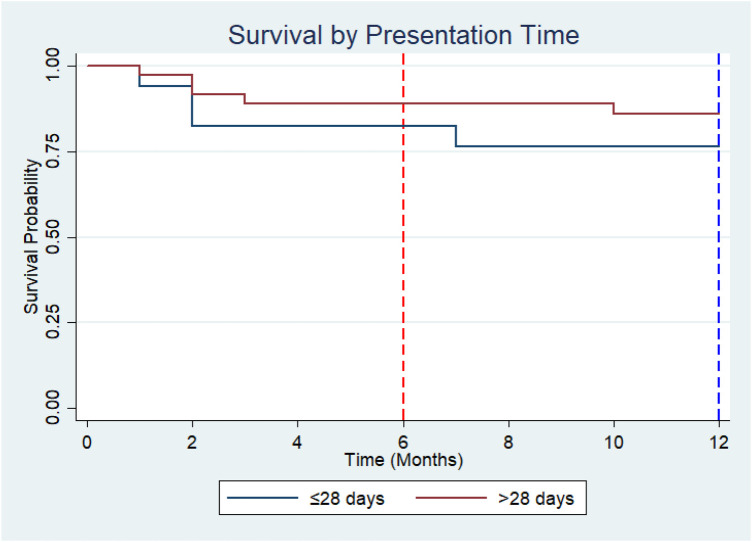
Kaplan–Meier survival curves for ALL patients stratified by presentation time.

Although children who presented after 28 days demonstrated slightly higher survival probabilities at both 6 and 12 months, the overlapping confidence intervals suggest that this difference was not statistically significant. This was confirmed by the log-rank test, which showed no significant difference in survival distributions between groups (*χ*^2^ = 0.79, *p* = 0.37) (Figure 4).

### Survival analysis by time to diagnosis

**Figure 5:**
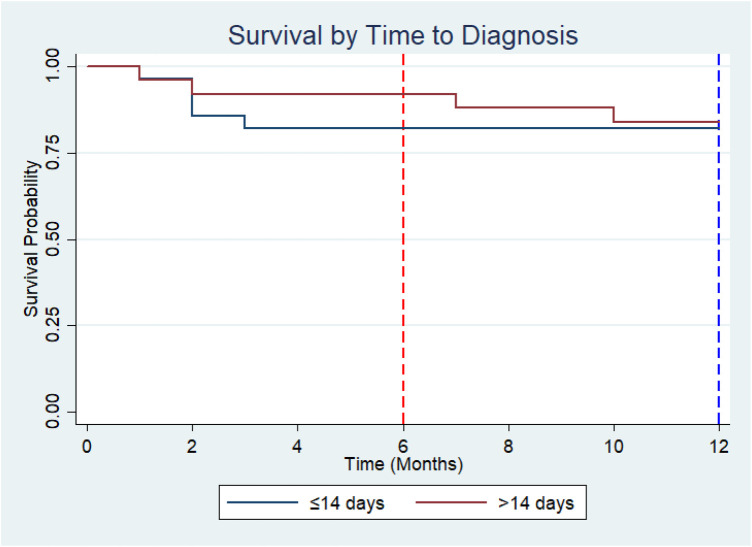
Kaplan–Meier survival curves for ALL patients stratified by time to diagnosis.

Kaplan–Meier survival estimates stratified by time to diagnosis showed that survival probability remained above 50% throughout the 12-month follow-up period in both groups; therefore, the median survival time was not reached for either group. At 6 months, the survival probability was 82.1% (95% CI: 62.3%–92.2%) among children diagnosed within 14 days of admission, compared with 92.0% (95% CI: 71.6%–97.9%) among those diagnosed after 14 days. At 12 months, survival probability remained 82.1% (95% CI: 62.3%–92.2%) in the ≤14-day group, while it declined modestly to 84.0% (95% CI: 62.8%–93.7%) in the >14-day group. Although children with longer time to diagnosis demonstrated slightly higher survival at 6 months, and comparable survival at 12 months, the overlapping confidence intervals indicate that these differences were not statistically significant. This was confirmed by the log-rank test, which showed no significant difference in survival distributions between the two groups (*χ*^2^ = 0.04, *p* = 0.84) (Figure 5).

### Survival analysis by referral pathway length

**Figure 6:**
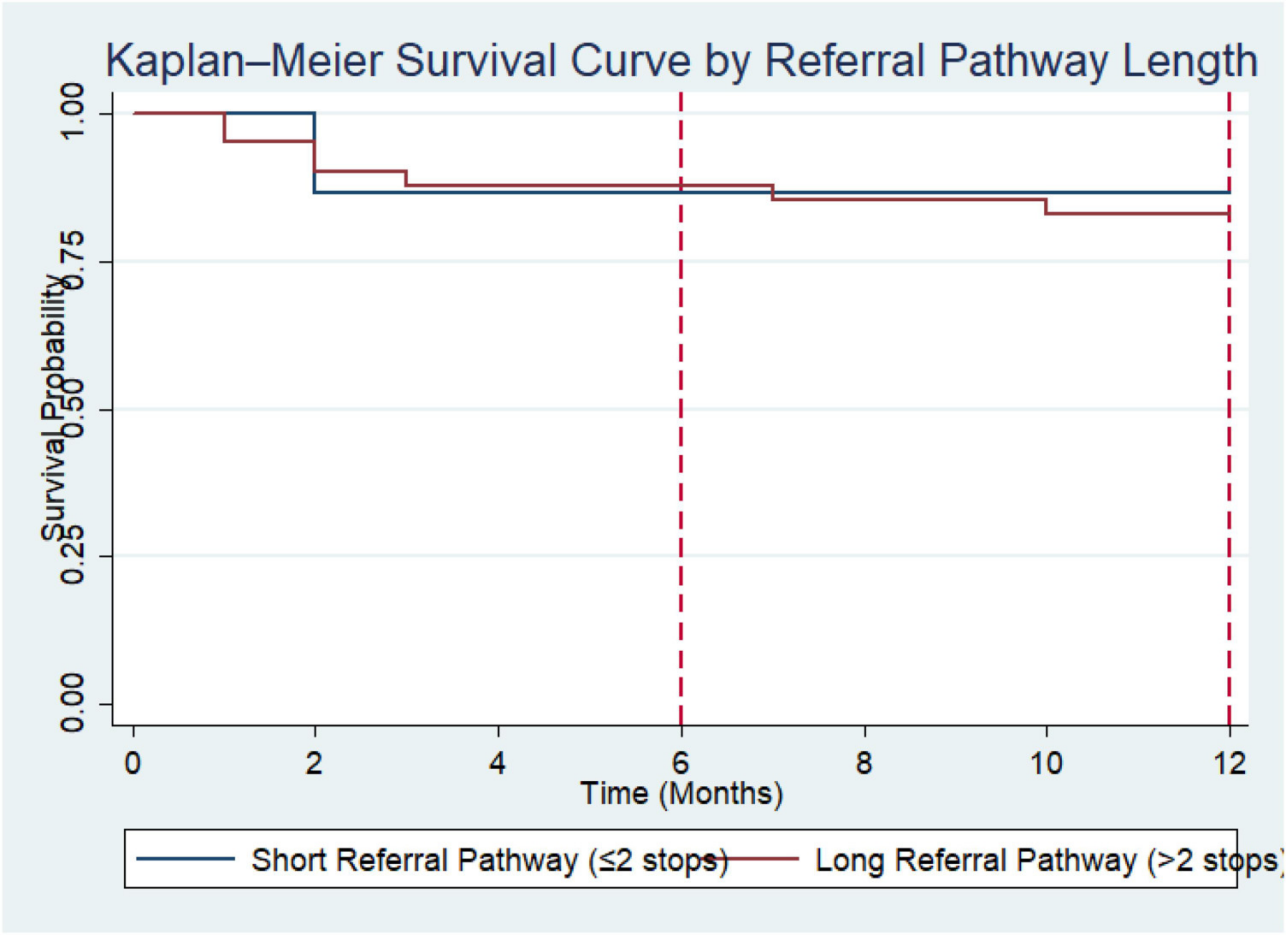
Kaplan–Meier survival curves for ALL patients stratified by pathways.

Kaplan–Meier survival estimates stratified by referral pathway length demonstrated that survival probability remained above 50% throughout the 12-month follow-up period in both groups; therefore, the median survival time was not reached for either short or long referral pathways. At 6 months, the survival probability was 86.7% (95% CI: 56.4%–96.5%) among children who followed a short referral pathway (≤2 health facilities visited before reaching MNH Oncology Unit), compared with 87.8% (95% CI: 73.2%–94.7%) among those who followed a long referral pathway (>2 health facilities visited before reaching MNH oncology unit). At 12 months, survival probability remained 86.7% (95% CI: 56.4%–96.5%) in the short pathway group, while it declined slightly to 82.9% (95% CI: 67.5%–91.5%) in the long pathway group. Although children who experienced shorter referral pathways showed marginally higher survival at 12 months, the confidence intervals overlapped substantially. The log-rank test showed no statistically significant difference in survival distributions between short and long referral pathways (*χ*^2^ = 0.11, *p* = 0.74), indicating that referral pathway length was not significantly associated with early survival in this cohort (Figure 6).

## Discussion

In this study, we explored patient pathways, presentation time, time to diagnosis, and survival outcome of pediatric ALL patients treated at the pediatric oncology unit of MNH, Dar es Salaam, Tanzania, between January and December 2024. The Tanzanian health system is structured into three levels: primary (dispensaries/health centers), secondary (regional referral hospitals), and tertiary (zonal and national hospitals). This study aimed to describe the care pathway of pediatric ALL patients within this system.

Findings showed that about 18.8% children presented directly to the MNH Oncology Unit via secondary-level facilities. This trend may be attributed to ongoing hospital-based childhood cancer awareness campaigns at referral hospitals in Dar es Salaam, as well as the impact of the national childhood cancer network in Tanzania, which has improved the prompt referral of suspected pediatric cancer cases directly to the MNH oncology unit. Half of the participants reported visiting primary-level health facilities (dispensaries, health centers, and district hospitals), accounting for 51.6%. Therefore, primary healthcare facilities should be a key target for facility-based health education programs on the early warning signs of pediatric cancers, as delays in the diagnosis of pediatric cancer cases are most likely to occur at this level of care. A study conducted in South India reported that pediatric cancer patients often enter the health system through mixed and fragmented pathways. The main entry point is decentralized, with many children presenting at non-specialized private facilities and low-level (primary) healthcare facilities, which significantly contribute to diagnostic and treatment delays (18). The findings of the present study are consistent with those reported at Bugando Medical Centre (BMC), where primary healthcare facilities were identified as the main entry point for most pediatric cancer patients into the health system, thereby playing a major role in contributing to delays (16). These observations suggest that primary healthcare facilities should be the primary target for interventions aimed at reducing both system inefficiencies and delays.

The time it takes for a child with ALL to present to a hospital after symptom onset can vary significantly. In this study, the median presentation time was 28.5 days, with an average of 40.7 days, indicating substantial postponement in seeking specialized care. This delay is shorter than that reported in a prospective cohort study from Pakistan, where many patients experienced delays exceeding 6 months before consulting an oncologist (7). Another study in South India revealed a median patient presentation time of 14 days (range: 1–90 days). These variations may reflect differences in health-seeking behavior, access to healthcare services, referral pathways, and caregiver awareness of childhood cancer symptoms. Nevertheless, the delay observed in our study remains clinically significant, as late presentation is a well-recognized contributor to advanced disease at diagnosis and poorer treatment outcomes in low- and middle-income countries (LMICs). Systemic and contextual factors contribute to delays in referral. Complex referral pathways within the healthcare system often result in prolonged time intervals before patients reach specialized centers for definitive diagnosis and treatment. Knowledge gaps among health workers regarding the clinical presentation of pediatric cancers at primary healthcare facilities contribute substantially to delayed recognition. The early symptoms of pediatric malignancies frequently overlap with common infectious and non-malignant conditions, which may lead to misdiagnosis or delayed referral to higher-level facilities.

Time to diagnosis is a key predictor of ALL patient outcomes. It refers to the interval between admission to an oncology center and receiving a definitive cancer diagnosis to initiate treatment. In this study, the median time to diagnosis was 14 days, with an average of 16 days. This is somewhat shorter than the findings of a study in South India, which reported a median diagnosis time of 41 days (range: 1–194 days) (18), and a study in Bangladesh, which reported a median diagnosis time of 60 days (15). Time to diagnosis can be influenced by parental or guardian awareness, sample acquisition, limited availability of advanced diagnostic resources, pathology facilities, and healthcare personnel's recognition of early warning signs of childhood cancer. In primary and secondary health facilities, these factors often contribute to prolonged time to confirmed diagnosis.

A 1-year survival analysis was conducted, showing that more than 50% of patients remained alive by the end of the observation period. The survival probability at 6 months was 79% (95% CI: 63%–88%), and at 12 months, it was 72% (95% CI: 55%–84%). Recent evidence indicates that the 1-year overall survival rate for pediatric patients with acute lymphoblastic leukemia (ALL) in HICs is very high, typically above 95%. This reflects excellent access to diagnosis, supportive care, strong referral systems, and early presentation time (13). Although most studies focus on 5-year survival (which exceeds 90% in HICs), registry data show that the mortality rate during the first year of diagnosis is low, often less than 5%. A global comparison of pediatric ALL outcomes noted that the 5-year survival rate in HICs is greater than 90%–95% (13), compared with approximately 67.8%–69.9% in LMICs (7). The 12-month survival observed in our study (72%) is substantially higher than that reported in South-West Uganda, where a 12-month survival rate of 42.5% among children with ALL has been documented (19). This difference may be attributed to variations in health system capacity, access to timely diagnosis and treatment, and supportive care services, despite both settings being within the LMIC context. According to the World Health Organization (2023), a global target has been set to achieve a 60% overall survival rate for children with cancer by 2030, with the aim of narrowing the outcome gap between HICs and LMICs (5, 20). At MNH, the majority of the deaths occurred during the induction phase, primarily due to severe infections and bleeding complications, consistent with previous reports (12). A smaller proportion of deaths occurred during the maintenance phase, often associated with disease relapse.

These findings highlight substantial delays in presentation and diagnosis despite relatively favorable short-term survival among children with ALL. There is a clear need for system-level policy and practice interventions to improve childhood ALL outcomes in Tanzania. Strengthening early detection and referral systems through national guidelines, enhanced training of primary and secondary healthcare providers, and community-based awareness initiatives could promote earlier health-seeking behavior and timely referral. Improving diagnostic capacity and patient flow within secondary and tertiary facilities, including fast-tracking suspected pediatric cancer cases at MNH, may reduce both prehospital and intrahospital delays.

### Study strengths and limitations

The inclusion of multiple data sources (hospital registries, clinical records, and caregiver follow-up) enhanced data completeness, and the use of Kaplan–Meier analysis enabled robust estimation of short-term survival. However, the retrospective single-center design, relatively small sample size, and short follow-up period, limited to 6 to 12 months, represent important limitations that may have reduced the power to detect subgroup differences and precluded assessment of long-term outcomes. Moreover, reliance on routinely collected data may have introduced recall and documentation biases, and the findings may not be fully generalizable beyond specialized referral settings. Notably, given the limited number of observed events within the defined follow-up period, inclusion of multiple additional covariates risked model overfitting and unstable estimates. The absence of clinically important variables—such as treatment adherence, potential interruptions in therapy due to drug stock-outs, co-morbid conditions such as severe malnutrition or concurrent infections, and detailed leukemia immunophenotypic and risk stratification categories (e.g., T-cell vs. B-cell lineage; high-risk vs. standard-risk ALL)—may therefore result in residual confounding, and findings should be interpreted within this context.

## Conclusion

The majority of patients admitted to the MNH pediatric oncology unit were referred from secondary-level healthcare facilities, which can be attributed to the presence of pediatricians at these secondary facilities. Half of the patients reported their first visit to primary-level facilities since the onset of initial leukemia symptoms. Significant late presentations and diagnostic delays were observed, which may contribute to declining survival over time. Referral pathways should be strengthened through (1) targeted training of primary healthcare workers on early warning signs of childhood cancer; (2) implementation of simplified national referral algorithms for suspected pediatric malignancy; (3) use of structured referral documentation tools; and (4) utilization of teleconsultation support linking primary facilities to tertiary oncology centers, ensuring a quick transition to specialized cancer care units. In addition, childhood cancer awareness campaigns should be directed at both the public and healthcare providers. Promoting universal health insurance coverage, investing in healthcare capacity, and fostering collaborations between HICs and LMICs are essential to sharing knowledge and resources, thus narrowing the survival gap.

## Data Availability

The raw data supporting the conclusions of this article will be made available by the authors, without undue reservation.
